# On the Signless Laplacian Spectral Radius of Bicyclic Graphs with Perfect Matchings

**DOI:** 10.1155/2014/374501

**Published:** 2014-06-11

**Authors:** Jing-Ming Zhang, Ting-Zhu Huang, Ji-Ming Guo

**Affiliations:** ^1^School of Mathematical Sciences, University of Electronic Science and Technology of China, Chengdu, Sichuan 611731, China; ^2^College of Science, China University of Petroleum, Shandong, Qingdao 266580, China; ^3^College of Science, East China University of Science and Technology, Shanghai 200237, China

## Abstract

The graph with the largest signless Laplacian spectral radius among all bicyclic graphs with perfect matchings is determined.

## 1. Introduction

Let *G* = (*V*, *E*) be a simple connected graph with vertex set *V* = {*v*_1_, *v*_2_,…, *v*_*n*_} and edge set *E*. Its adjacency matrix *A*(*G*) = (*a*_*ij*_) is defined as *n* × *n* matrix (*a*_*ij*_), where *a*_*ij*_ = 1 if *v*_*i*_ is adjacent to *v*_*j*_, and *a*_*ij*_ = 0, otherwise. Denote by *d*(*v*_*i*_) or *d*_*G*_(*v*_*i*_) the degree of the vertex *v*_*i*_. Let *Q*(*G*) = *D*(*G*) + *A*(*G*) be the signless Laplacian matrix of graph *G*, where *D*(*G*) = diag⁡(*d*(*v*_1_), *d*(*v*_2_),…, *d*(*v*_*n*_)) denotes the diagonal matrix of vertex degrees of *G*. It is well known that *A*(*G*) is a real symmetric matrix and *Q*(*G*) is a positive semidefinite matrix. The largest eigenvalues of *A*(*G*) and *Q*(*G*) are called the spectral radius and the signless Laplacian spectral radius of *G*, denoted by *ρ*(*G*) and *q*(*G*), respectively. When *G* is connected, *A*(*G*) and *Q*(*G*) are a nonnegative irreducible matrix. By the well-known Perron-Frobenius theory, *ρ*(*G*) is simple and has a unique positive unit eigenvector and so does *q*(*G*). We refer to such an eigenvector corresponding to *q*(*G*) as the Perron vector of *G*.

Two distinct edges in a graph *G* are independent if they are not adjacent in *G*. A set of mutually independent edges of *G* is called a matching of *G*. A matching of maximum cardinality is a maximum matching in *G*. A matching *M* that satisfies 2 | *M* | = *n* = |*V*(*G*)| is called a perfect matching of the graph *G*. Denote by *C*_*n*_ and *P*_*n*_ the cycle and the path on *n* vertices, respectively.

The characteristic polynomial of *A*(*G*) is det⁡(*xI* − *A*(*G*)), which is denoted by Φ(*G*) or Φ(*G*, *x*). The characteristic polynomial of *Q*(*G*) is det⁡(*xI* − *Q*(*G*)), which is denoted by Ψ(*G*) or Ψ(*G*, *x*).

**Figure 1:**
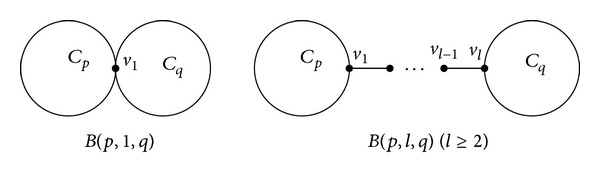
*B*(*p*, 1, *q*) and *B*(*p*, *l*, *q*)  (*l* ≥ 2).

**Figure 2:**
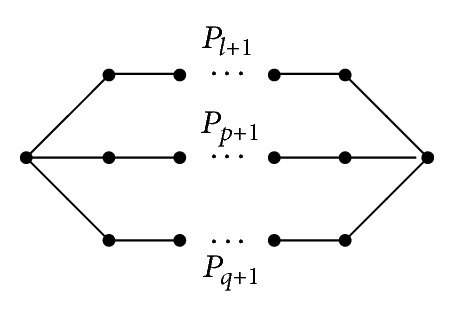
*P*(*p*, *l*, *q*).

A bicyclic graph is a connected graph in which the number of vertices equals the number of edges minus one. Let *C*_*p*_ and *C*_*q*_ be two vertex-disjoint cycles. Suppose that *v*_1_ is a vertex of *C*_*p*_ and *v*_*l*_ is a vertex of *C*_*q*_. Joining *v*_1_ and *v*_*l*_ by a path *v*_1_ *v*_2_ ⋯ *v*_*l*_ of length *l* − 1, where *l* ≥ 1 and *l* = 1 means identifying *v*_1_ with *v*_*l*_, denoted by *B*(*p*, *l*, *q*), is called an *∞*-graph (see Figure 1). Let *P*_*l*+1_, *P*_*p*+1_, and *P*_*q*+1_ be the three vertex-disjoint paths, where *l*, *p*, *q* ≥ 1, and at most one of them is 1. Identifying the three initial vertices and the three terminal vertices of them, respectively, denoted by *P*(*l*, *p*, *q*), is called a *θ*-graph (see Figure 2).

Let *B*_*n*_(2*μ*) be the set of all bicyclic graphs on *n* = 2*μ*  (*μ* ≥ 2) vertices with perfect matchings. Obviously *B*_*n*_(2*μ*) consists of two types of graphs: one type, denoted by *B*_*n*_^+^(2*μ*), is a set of graphs each of which is an *∞*-graph with trees attached; the other type, denoted by *B*_*n*_^++^(2*μ*), is a set of graphs each of which is *θ*- graph with trees attached. Then we have *B*_*n*_(2*μ*) = *B*_*n*_^+^(2*μ*) ∪ *B*_*n*_^++^(2*μ*).

The investigation on the spectral radius of graphs is an important topic in the theory of graph spectra, in which some early results can go back to the very beginnings (see [1]). The recent developments on this topic also involve the problem concerning graphs with maximal or minimal spectral radius of a given class of graphs. In [2], Chang and Tian gave the first two spectral radii of unicyclic graphs with perfect matchings. Recently, Yu and Tian [3] gave the first two spectral radii of unicyclic graphs with a given matching number; Guo [4] gave the first six spectral radii over the class of unicyclic graphs on a given number of vertices; and Guo [5] gave the first ten spectral radii over the class of unicyclic graphs on a given number of vertices and the first four spectral radii of unicyclic graphs with perfect matchings. For more results on this topic, the reader is referred to [6–9] and the references therein.

In this paper, we deal with the extremal signless Laplacian spectral radius problems for the bicyclic graphs with perfect matchings. The graph with the largest signless Laplacian spectral radius among all bicyclic graphs with perfect matchings is determined.

## 2. Lemmas

Let *G* − *u* or *G* − *uv* denote the graph obtained from *G* by deleting the vertex *u* ∈ *V*(*G*) or the edge *uv* ∈ *E*(*G*). A pendant vertex of *G* is a vertex with degree 1. A path *P* : *vv*_1_ *v*_2_ ⋯ *v*_*k*_ in *G* is called a pendant path if *d*(*v*_1_) = *d*(*v*_2_) = ⋯ = *d*(*v*_*k*−1_) = 2 and *d*(*v*_*k*_) = 1. If *k* = 1, then we say *vv*_1_ is a pendant edge of the graph *G*.

In order to complete the proof of our main result, we need the following lemmas.

**Lemma 1 (see [10, 11])**

Let *G* be a connected graph and *u*, *v* two vertices of *G*. Suppose that *v*_1_, *v*_2_,…, *v*_*s*_ ∈ *N*(*v*)∖{*N*(*u*) ∪ *u*}  (1 ≤ *s* ≤ *d*(*v*)) and *x* = (*x*_1_, *x*_2_,…, *x*_*n*_) is the Perron vector of *G*, where *x*_*i*_ corresponds to the vertex *v*_*i*_  (1 ≤ *i* ≤ *n*). Let G* be the graph obtained from *G* by deleting the edges *vv*_*i*_ and adding the edges *uv*_*i*_  (1 ≤ *i* ≤ *s*). If *x*_*u*_ ≥ *x*_*v*_, then *q*(*G*) < *q*(G*).

The cardinality of a maximum matching of *G* is commonly known as its matching number, denoted by *μ*(*G*).

From Lemma 1, we have the following results.

**Corollary 2**

Let *w* and *v* be two vertices in a connected graph *G* and suppose that *s* paths {*ww*_1_ *w*_1_′, *ww*_2_ *w*_2_′,…, *ww*_*s*_ *w*_*s*_′} of length 2 are attached to *G* at *w* and *t* paths {*vv*_1_ *v*_1_′, *vv*_2_ *v*_2_′,…, *vv*_*t*_ *v*_*t*_′} of length 2 are attached to *G* at *v* to form *G*_*s*,*t*_. Then either *q*(*G*_*s*+*i*,*t*−*i*_) > *q*(*G*_*s*,*t*_)  (1 ≤ *i* ≤ *t*) or *q*(*G*_*s*−*i*,*t*+*i*_) > *q*(*G*_*s*,*t*_)  (1 ≤ *i* ≤ *s*)or *μ*(*G*_0,*s*+*t*_) = *μ*(*G*_*s*+*t*,0_) = *μ*(*G*_*s*,*t*_).

**Corollary 3**

Suppose *u* is a vertex of graph *G* with *d*(*u*) ≥ 2. Let *G* : *uv* be a graph obtained by attaching a pendant edge *uv* to *G* at *u*. Suppose *t* paths {*vv*_1_ *v*_1_′,…, *vv*_*t*_ *v*_*t*_′} of length 2 are attached to *G* : *uv* at *v* to form *L*_0,*t*_. Let

(1) $$\begin{array}{c} M_{1,t}=L_{0,t}-vv_{1}-\cdots -vv_{t}+uv_{1}+\cdots +uv_{t}. \end{array}$$

If *L*_0,*t*_ has a perfect matching, then we have that *M*_1,*t*_ has a perfect matching and

(2) $$\begin{array}{c} q\left(M_{1,t}\right)>q\left(L_{0,t}\right),\;\left(t\geq 1\right). \end{array}$$

An internal path of a graph *G* is a sequence of vertices *v*_1_, *v*_2_,…, *v*_*m*_ with *m* ≥ 2 such that

1. the vertices in the sequences are distinct (except possibly *v*_1_ = *v*_*m*_);
2. *v*_*i*_ is adjacent to *v*_*i*+1_, (*i* = 1,2,…, *m* − 1);
3. the vertex degrees *d*(*v*_*i*_) satisfy *d*(*v*_1_) ≥ 3, *d*(*v*_2_) = ⋯ = *d*(*v*_*m*−1_) = 2 (unless *m* = 2) and *d*(*v*_*m*_) ≥ 3.

Let *G* be a connected graph, and *uv* ∈ *E*(*G*). The graph *G*_*uv*_ is obtained from *G* by subdividing the edge *uv*, that is, adding a new vertex *w* and edges *uw*, *wv* in *G* − *uv*. By similar reasoning as that of Theorem 3.1 of [12], we have the following result.

**Lemma 4**

Let *P* : *v*_1_ *v*_2_ ⋯ *v*_*k*_  (*k* ≥ 2) be an internal path of a connected graph *G*. Let *G*′ be a graph obtained from *G* by subdividing some edge of *P*. Then we have *q*(*G*′) < *q*(*G*).

**Corollary 5**

Suppose that *v*_1_ *v*_2_ ⋯ *v*_*k*_  (*k* ≥ 3) is an internal path of the graph *G* and *v*_1_ *v*_*k*_ ∉ *E*(*G*) for *k* = 3. Let G* be the graph obtained from *G* − *v*_*i*_ *v*_*i*+1_ − *v*_*i*+1_ *v*_*i*+2_  (1 ≤ *i* ≤ *k* − 2) by amalgamating *v*_*i*_, *v*_*i*+1_, and *v*_*i*+2_ to form a new vertex *w*_1_ together with attaching a new pendant path *w*_1_ *w*_2_ *w*_3_ of length 2 at *w*_1_. Then *q*(G*) > *q*(*G*) and *μ*(G*) ≥ *μ*(*G*).

**Proof**

From Lemma 4 and the well-known Perron-Frobenius theorem, It is easy to prove that *q*(G*) > *q*(*G*). Next, we prove that *μ*(G*) ≥ *μ*(*G*). Let *M* be a maximum matching of *G*. If *v*_*i*_ *v*_*i*+1_ ∈ *M* or *v*_*i*+1_ *v*_*i*+2_ ∈ *M*, then {*M* − {*v*_*i*_ *v*_*i*+1_}}∪{*w*_2_ *w*_3_} or {*M* − {*v*_*i*+1_ *v*_*i*+2_}}∪{*w*_2_ *w*_3_} is a matching of G*. Thus, *μ*(G*) ≥ *μ*(*G*); If *v*_*i*_ *v*_*i*+1_ ∉ *M* and *v*_*i*+1_ *v*_*i*+2_ ∉ *M*, then there exist two edges *v*_*i*_ *u* and *v*_*i*+2_ *v* ∈ *M*. Thus, {*M* − {*v*_*i*_ *u*}}∪{*w*_2_ *w*_3_} is a matching of G*. Hence, *μ*(G*) ≥ *μ*(*G*), completing the proof.

Let *S*(*G*) be the subdivision graph of *G* obtained by subdividing every edge of *G*.

**Lemma 6 (see [13, 14])**

Let *G* be a graph on *n* vertices and *m* edges, Φ(*G*) = det⁡(*xI* − *A*(*G*)), Ψ(*G*) = det⁡(*xI* − *Q*(*G*)). Then Φ(*S*(*G*)) = *x* ^*m*−*n*^Ψ(*G*, *x* ^2^).

**Lemma 7 (see [15])**

Let *u* be a vertex of a connected graph *G*. Let *G*_*k*,*l*_  (*k*, *l* ≥ 0) be the graph obtained from *G* by attaching two pendant paths of lengths *k* and *l* at *u*, respectively. If *k* ≥ *l* ≥ 1, then *q*(*G*_*k*,*l*_) > *q*(*G*_*k*+1,*l*−1_).

**Corollary 8**

Suppose that *v*_1_ *v*_2_ ⋯ *v*_*k*_  (*k* ≥ 3) is a pendant path of the graph *G* with *d*(*v*_1_) ≥ 3. Let G* be the graph obtained from *G* − *v*_1_ *v*_2_ − *v*_2_ *v*_3_ by amalgamating *v*_1_, *v*_2_, and *v*_3_ to form a new vertex *w*_1_ together with attaching a new pendant path *w*_1_ *w*_2_ *w*_3_ *of length *2 at *w*_1_. Then *q*(G*) > *q*(*G*) and *μ*(G*) ≥ *μ*(*G*).

**Proof**

By Lemma 7 we have *q*(G*) > *q*(*G*). By the proof as that of Corollary 5, we have *μ*(G*) ≥ *μ*(*G*).

**Lemma 9 (see [16])**

Let *e* = *uv* be an edge of *G*, and let *C*(*e*) be the set of all circuits containing *e*. Then Φ(*G*) satisfies

(3) $$\begin{array}{c} \Phi \left(G\right)=\Phi \left(G-e\right)-\Phi \left(G-u-v\right)-2\underset{Z}{\sum }\Phi \left(G-V\left(Z\right)\right), \end{array}$$

where the summation extends over all *Z* ∈ *C*(*e*).

**Lemma 10 (see [16])**

Let *v* be a vertex of *G*, and let *φ*(*v*) be the collection of circuits containing *v*, and let *V*(*Z*) denote the set of vertices in the circuit *Z*. Then the characteristic polynomial Φ(*G*) satisfies

(4) Φ(G)=xΦ(G−v)−∑wΦ(G−v−w) −2∑Z∈φ(v)Φ(G−V(Z)),

where the first summation extends over those vertices *w* adjacent to *v*, and the second summation extends over all *Z* ∈ *φ*(*v*).

**Lemma 11 (see [17])**

Let *G* be a connected graph, and let *G*′ be a proper spanning subgraph of *G*. Then *ρ*(*G*) > *ρ*(*G*′), and, for *x* ≥ *ρ*(*G*), Φ(*G*′) > Φ(*G*).

Let Δ(*G*) denote the maximum degree of *G*. From Lemma 11, we have $\rho (G)\geq \sqrt{\Delta (G)}$.

**Lemma 12 (see [13])**

Let *G* be a connected graph, and let *G*′ be a proper spanning subgraph of *G*. Then *q*(*G*) > *q*(*G*′).

**Lemma 13 (see [18])**

Let *G* = (*V*, *E*) be a connected graph with vertex set *V* = {*v*_1_, *v*_2_,…, *v*_*n*_}. Suppose that *v*_1_ *v*_2_ ∈ *E*(*G*), *v*_1_ *v*_3_ ∈ *E*(*G*), *v*_1_ *v*_4_ ∈ *E*(*G*), *d*(*v*_3_) ≥ 2, *d*(*v*_4_) ≥ 2, *d*(*v*_1_) = 3, and *d*(*v*_2_) = 1. Let *G*_*v*_1_*v*_3__(*G*_*v*_1_*v*_4__) be the graph obtained from *G* − *v*_1_ *v*_3_(*G* − *v*_1_ *v*_4_) by amalgamating *v*_1_ and *v*_3_(*v*_4_) to form a new vertex *w*_1_(*w*_3_) together with subdivising the edge *w*_1_ *v*_2_(*w*_3_ *v*_2_)* with a new vertex w*_2_(*w*_4_). If $q=q(G)>3+\sqrt{5}\approx 5.23606$, then

1. either *q*(*G*_*v*_1_*v*_3__) > *q*(*G*)* or q*(*G*_*v*_1_*v*_4__) > *q*(*G*);
2. *μ*(*G*_*v*_1_*v*_3__) ≥ *μ*(*G*)* and μ*(*G*_*v*_1_*v*_4__) ≥ *μ*(*G*).

**Lemma 14 (see [18])**

Suppose *u* is a vertex of the bicyclic graph *G* with *d*_*G*_(*u*) ≥ 2. Let *G* : *uv* be a graph obtained by attaching a pendant edge *uv* to *G* at *u*. Suppose that a pendant edge *vw*_1_ and *t* paths {*vv*_1_ *v*_1_′,…, *vv*_*t*_ *v*_*t*_′} of length 2 are attached to *G* : *uv* at *v* to form *L*_1,*t*_. Let *M*_0,*t*+1_ = *L*_1,*t*_ − *vv*_1_ − ⋯−*vv*_*t*_ + *uv*_1_ + ⋯+*uv*_*t*_. Then we have

1. *q*(*M*_0,*t*+1_) > *q*(*L*_1,*t*_), (*t* ≥ 1);
2. *μ*(*L*_1,*t*_) ≤ *μ*(*M*_0,*t*+1_).

## 3. Main Results

**Lemma 15**

**Figure 3:**
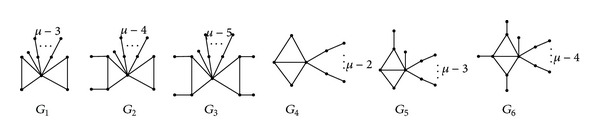
*G*_1_–*G*_6_.

Let *G*_1_, *G*_2_,…, *G*_6_ be the graphs as Figure 3. Then for *μ* ≥ 3, we have *q*(*G*_1_) > *q*(*G*_*i*_), (*i* = 2,3,…, 6).

**Proof**

From Lemma 10, we have

(5) Φ(S(G1)) =x(x2−1)(x4−3x2+1)μ−3(x5−4x3+3x)2  −(μ−3)(x2−1)(x3−2x)  ×(x4−3x2+1)μ−4(x5−4x3+3x)2  −x(x4−3x2+1)μ−3(x5−4x3+3x)2  −4(x2−1)(x4−3x2+1)μ−3  ×(x5−4x3+3x)(x4−3x2+2)Φ(S(G2))=x(x2−1)(x4−3x2+1)μ−4(x5−4x3+3x) ×(x9−8x7+19x5−14x3+3x) −(μ−4)(x2−1)(x3−2x)(x4−3x2+1)μ−5 ×(x5−4x3+3x)(x9−8x7+19x5−14x3+3x) −x(x4−3x2+1)μ−4(x5−4x3+3x) ×(x9−8x7+19x5−14x3+3x)−2(x2−1) ×(x4−3x2+1)μ−3 ×(x9−8x7+19x5−14x3+3x) −2(x2−1)(x4−3x2+1)μ−4(x5−4x3+3x) ×(x8−7x6+14x4−8x2+1)−2(x2−1) ×(x4−3x2+1)μ−4 ×(x9−8x7+19x5−14x3+3x) −2(x2−1)3(x4−3x2+1)μ−4(x5−4x3+3x).

From (5), we have

(6) Φ(S(G2))−Φ(S(G1))=x3(x4−3x2+1)μ−5×[(−2+μ)x14+(22−10μ)x12+(−97+39μ)x10+(221−75μ)x8+(−278+74μ)x6+(189−35μ)x4+(−63+6μ)x2+8].

If *μ* ≥ 12, for $x\geq \rho (S(G_{1}))\geq \sqrt{\Delta S(G_{1})}=\sqrt{\mu +2}$, it is easy to prove that Φ(*S*(*G*_2_)) − Φ(*S*(*G*_1_)) > 0. Hence, *ρ*(*S*(*G*_1_)) > *ρ*(*S*(*G*_2_)) for *μ* ≥ 12. When *μ* = 4,5,…, 11, by direct calculation, we also get *ρ*(*S*(*G*_1_)) > *ρ*(*S*(*G*_2_)), respectively. So, *ρ*(*S*(*G*_1_)) > *ρ*(*S*(*G*_2_)) for *μ* ≥ 4. By Lemma 6, we know that $\rho (S(G))=\sqrt{q(G)}$. Hence, *q*(*G*_1_) > *q*(*G*_2_)  (*μ* ≥ 4). By similar method, the result is as follows.

**Theorem 16**

If *G* ∈ *B*_*n*_(2*μ*)  (*n* ≥ 6), then *q*(*G*) ≤ *q*(*G*_1_), with equality if and only if *G* = *G*_1_.

**Proof**

Let *X* = (*x*_1_, *x*_2_,…, *x*_*n*_)^*T*^ be the Perron vector of *G*. From Lemma 12 and by direct calculations, we have, for *μ* ≥ 3, $q(G_{1})>q(B(3,1,3))\approx 5.5615>3+\sqrt{5}$. So, in the following, we only consider those graphs, which have signless Laplacian spectral radius greater than $q(G)>3+\sqrt{5}$.

Choose G* ∈ *B*_*n*_(2*μ*) such that *q*(G*) is as large as possible. Then G* consists of a subgraph *H* which is one of graphs *B*(*p*, 1, *q*), *B*(*p*, *l*, *q*), and *P*(*p*, *l*, *q*) (see Figures 1 and 2).

Let *T* be a tree attached at some vertex, say, *z*, of *H*; |*V*(*T*)| is the number of vertices of *T* including the vertex *z*. In the following, we prove that tree *T* is formed by attaching at most one path of length 1 and other paths of length 2 at *z*.

Suppose *P* : *v*_0_ *v*_1_ ⋯ *v*_*k*_ is a pendant path of G* and *v*_*k*_ is a pendant vertex. If *k* ≥ 3, let *H*_1_ = G* − *v*_2_ *v*_3_ + *v*_0_ *v*_3_. From Corollary 8, we have *H*_1_ ∈ *B*_*n*_(2*μ*) and *q*(*H*_1_) > *q*(G*), which is a contradiction.

For each vertex *u* ∈ *V*(*T* − *z*), we prove that *d*(*u*) ≤ 2. Otherwise, there must exist some vertex *u*_0_ of *T* − *z* such that *d*(*z*, *u*_0_) = max⁡{*d*(*z*, *v*) | *v* ∈ *V*(*T*) − *z*, *d*(*v*) ≥ 3}. From the above proof, we have the pendant paths attached *u*_0_ which have length of at most 2. Obviously, there exists an internal path between *u*_0_ and some vertex *w* of G*, denoted by $\bar{P}:u_{0}w_{1}\cdots w_{m}\;(w_{m}=w)$. If *m* ≥ 2, let *H*_2_ be the graph obtained from G* − *u*_0_ *w*_1_ − *w*_1_ *w*_2_ by amalgamating *u*_0_, *w*_1_, and *w*_2_ to form a new vertex *s*_1_ together with attaching a new pendant path *s*_1_ *s*_2_ *s*_3_ of length 2 at *s*_1_. From Corollary 5, we have *H*_2_ ∈ *B*_*n*_(2*μ*) and *q*(*H*_2_) > *q*(G*), which is a contradiction. If *m* = 1, by Lemma 14 and Corollary 3, we can get a new graph *H*_3_ such that *H*_3_ ∈ *B*_*n*_(2*μ*) and *q*(*H*_3_) > *q*(G*), which is a contradiction.

From the proof as above, we have the tree *T* which is obtained by attaching some pendant paths of length 2 and at most one pendant path of length 1 at *z*.

From Corollary 2, we have all the pendant paths of length 2 in G* which must be attached at the same vertex of *H*.

In the following, we prove that G* is isomorphic to one of graphs *G*_1_, *G*_2_,…, *G*_6_ (see Figure 3). We distinguish the following two cases:

Case  1 (G* ∈ *B*_*n*_^+^(2*μ*)). We prove that G* is isomorphic to one of graphs *G*_1_, *G*_2_, and *G*_3_.

Assume that there exists some cycle *C*_*p*_ of G* with length of at least 4. From Corollary 5, we have each internal path of G*, which is not a triangle, has length 1. Note that all the pendant paths of length 2 in G* must be attached at the same vertex, then there must exist edges *v*_1_ *v*_2_ ∈ *E*(G*), *v*_1_ *v*_3_ ∈ *E*(*C*_*p*_), and *v*_1_ *v*_4_ ∈ *E*(*C*_*p*_) and *d*(*v*_1_) = 3, *d*(*v*_2_) = 1, *d*(*v*_3_) ≥ 3, and *d*(*v*_4_) ≥ 3. Let *H*_4_  (*H*_5_) be the graph obtained from G* − *v*_1_ *v*_3_  (G* − *v*_1_ *v*_4_) by amalgamating *v*_1_ and *v*_3_(*v*_4_) to form a new vertex *y*_1_(*y*_3_) together with subdividing the edge *y*_1_ *v*_2_  (*y*_3_ *v*_2_) with a new vertex *y*_2_  (*y*_4_). From Lemma 13, we have *H*_*i*_ ∈ *B*_*n*_^+^(2*μ*)  (*i* = 4,5) and either *q*(*H*_4_) > *q*(G*) or *q*(*H*_5_) > *q*(G*), which is a contradiction. Then for each cycle *C*_*g*_ of G*, we have *g* = 3.

Assume that *l* ≥ 4. If there exists an internal path ${\bar{P}}^{*}:v_{i}v_{i+1}\cdots v_{m}\;(1\leq i<m\leq l)$ with length greater than 1 in G*. Then, by Corollary 5, we can get a new graph *H*_6_ such that *q*(*H*_6_) > *q*(G*) and *H*_6_ ∈ *B*_*n*_^+^(2*μ*), which is a contradiction. Thus, *d*(*v*_*i*_) ≥ 3  (*i* = 1,2,…, *l*) and either *d*(*v*_2_) = 3 or *d*(*v*_3_) = 3. By Lemma 13, we can also get a new graph *H*_7_ such that *q*(*H*_7_) > *q*(G*) and *H*_7_ ∈ *B*_*n*_^+^(2*μ*), which is a contradiction. Hence, *l* ≤ 3.

**Figure 4:**
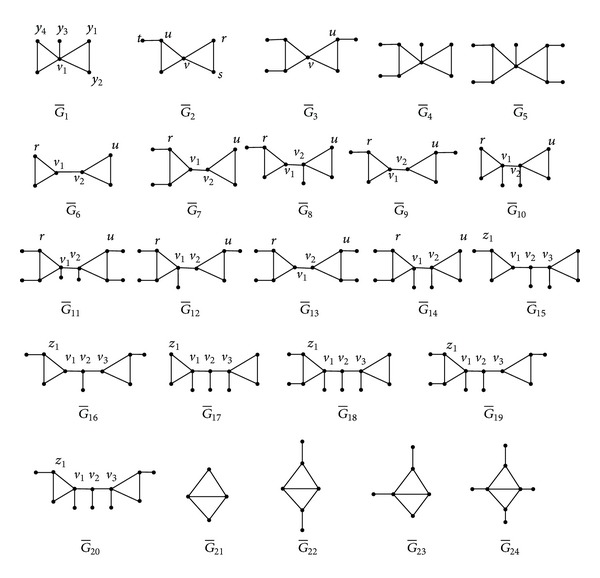
${\bar{G}}_{1}\text{–}{\bar{G}}_{24}$.

We distinguish the following three subcases:

*Subcase  1.1 (l* = 1). Then G* is the graph obtained by attaching all the pendant paths of length 2 at the same vertex of $\bar{G}$, where $\bar{G}$ is one of graphs ${\bar{G}}_{1},\ldots ,{\bar{G}}_{5}$ (see Figure 4).

Assume that $\bar{G}={\bar{G}}_{2}$. If *x*_*u*_ ≥ *x*_*v*_, let *H*_8_ = G* − *rv* − *sv* + *ru* + *su*; if *x*_*v*_ ≥ *x*_*u*_, let *H*_9_ = G* − *ut* + *tv*. Obviously, *H*_*i*_ ∈ *B*_*n*_^+^(2*μ*)  (*i* = 8,9) and either *q*(*H*_8_) > *q*(G*) or *q*(*H*_9_) > *q*(G*) by Lemma 1, which is a contradiction. By similar reasoning, we have also $\bar{G}\neq {\bar{G}}_{3}$.

*Subcase  1.2 (l* = 2). Then G* is the graph obtained by attaching all the pendant paths of length 2 at the same vertex of $\bar{G}$, where $\bar{G}$ is one of graphs ${\bar{G}}_{6},\ldots ,{\bar{G}}_{14}$ (see Figure 4).

Assume that $\bar{G}={\bar{G}}_{6}$. If *x*_*v*_1__ ≥ *x*_*v*_2__, let *H*_10_ = G* − *v*_2_ *u* + *v*_1_ *u*; if *x*_*v*_2__ ≥ *x*_*v*_1__, let *H*_11_ = G* − *v*_1_ *r* + *v*_2_ *r*. Obviously, *H*_*i*_ ∈ *B*_*n*_^+^(2*μ*)  (*i* = 10,11) and either *q*(*H*_10_) > *q*(G*) or *q*(*H*_11_) > *q*(G*) by Lemma 1, which is a contradiction. By similar reasoning, we have also $\bar{G}\neq {\bar{G}}_{j}\;(j=6,\ldots ,14)$.

*Subcase  1.3 (l* = 3). Then G* is the graph obtained by attaching all the pendant paths of length 2 at the same vertex of $\bar{G}$, where $\bar{G}$ is one of graphs ${\bar{G}}_{15},\ldots ,{\bar{G}}_{20}$ (see Figure 4).

Assume that $\bar{G}={\bar{G}}_{15}$. If *x*_*v*_1__ ≥ *x*_*v*_2__, let *H*_12_ = G* − *v*_2_ *v*_3_ + *v*_1_ *v*_3_; if *x*_*v*_2__ ≥ *x*_*v*_1__, let *H*_13_ = G* − *v*_1_ *z*_1_ + *v*_2_ *z*_1_. Obviously, *H*_*i*_ ∈ *B*_*n*_^+^(2*μ*)  (*i* = 12,13) and either *q*(*H*_12_) > *q*(G*) or *q*(*H*_13_) > *q*(G*) by Lemma 1, a contradiction. By similar reasoning, we have also $\bar{G}\neq {\bar{G}}_{j}\;(j=15,\ldots ,20)$.

Thus, $\bar{G}$ is isomorphic to one of the graphs ${\bar{G}}_{1}$, ${\bar{G}}_{4}$ and ${\bar{G}}_{5}$. In the following, we prove that G* is isomorphic to one of graphs *G*_1_, *G*_2_ and *G*_3_.

Assume that G* is obtained by attaching all the pendant paths of length 2 at vertex *y*_4_ of ${\bar{G}}_{1}$. If *x*_*v*_1__ ≥ *x*_*y*_4__, let *H*_14_ be the graph obtained from ${\bar{G}}_{1}$ by attaching *μ* − 3 pendant paths of length 2 at *v*_1_. If *x*_*y*_4__ ≥ *x*_*v*_1__, let *H*_15_ = G* − *v*_1_ *y*_3_ − *v*_1_ *y*_1_ − *v*_1_ *y*_2_ + *y*_4_ *y*_3_ + *y*_4_ *y*_1_ + *y*_4_ *y*_2_. Obviously, *H*_14_ = *H*_15_ = *G*_1_ and *q*(*G*_1_) > *q*(G*) by Lemma 1, a contradiction. Then G* = *G*_1_. By similar reasoning, the result follows.

Case  2 (G* ∈ *B*_*n*_^++^(2*μ*)). By similar reasoning as that of Case  1, we have G* is the graph obtained by attaching all the pendant paths of length 2 at the same vertex of $\bar{G}$, where $\bar{G}$ is one of graphs ${\bar{G}}_{21},\ldots ,{\bar{G}}_{24}$ (see Figure 4).

From Lemma 1, it is easy to prove that $\bar{G}\neq {\bar{G}}_{22}$ and all the pendant paths of length 2 are attached at the vertex of degree 3 of ${\bar{G}}_{21}$ or of degree 4 of ${\bar{G}}_{i}\;(i=23,24)$. Thus, G* is isomorphic to one of graphs *G*_4_, *G*_5_ and *G*_6_ (see Figure 3).

So, G* is isomorphic to one of graphs *G*_1_,…, *G*_6_. From Lemma 15, we know *q*(*G*_1_) > *q*(*G*_*i*_), (*i* = 2,3,…, 6). Thus, G* = *G*_1_.
